# Supplementary material for: Predicting lung aging using scRNA-Seq data
Source: PLoS Comput Biol. 2024 Dec 19;20(12):e1012632. doi: 10.1371/journal.pcbi.1012632 (PMC11741621; doi:10.1371/journal.pcbi.1012632)
Supplement: S1 Text — (DOCX) [file pcbi.1012632.s001.docx]

# **Supplementary Information**

Identification of cell types and key genes for lung aging from large single cell atlas

Qi Song^1^, Alex Singh^1^, John McDonough, Naftali Kaminski, Ziv Bar-Joseph^12*^

1Computational Biology Department, School of Computer Science, Carnegie Mellon University, Pittsburgh, PA 15213, USA

2Machine Learning Department, School of Computer Science, Carnegie Mellon University, Pittsburgh, PA 15213, USA

* To whom correspondence should be addressed. Tel: 412-2688595; Email: zivbj@cs.cmu.edu

**MATERIALS AND METHODS**

**Dataset Preprocessing**

We performed several steps to filter cells and genes in these datasets: 1) for all datasets, genes expressed in less than 3 cells and cells expressing less than 100 genes were removed; 2) donors without age information were also removed; 3) we removed cells annotated as multiplets; 4) To ensure consistency across different datasets, we removed cells from tissues that were not included in all 4 datasets (See S1 Text and S1 Fig for details).

To ensure consistency across different datasets, we removed the following tissues and cells in the HLCA dataset: nose tissue, cells from lung trachea, cells from diseased donors, and cells annotated as the following HLCA cell types: Club (nasal), Goblet (nasal), Multiciliated (nasal), SMG serous (nasal).

**Overview of the datasets**

The distribution of donor ages are similar across these datasets (see S1 Fig). We noticed that there is a missing section of age distribution for the nuc-seq dataset (section of age 30~40 is missing, S1 Fig). We therefore removed the three donors with ages ≤ 30 to avoid outliers during modeling.

**Nuclear-seq data profiling**

Written informed consent of donors was not obtained as Belgian legislation stipulates donor lungs rejected for transplantation can be used for research (ethics committee University Hospitals Leuven, Belgium, institutional review board number S52174, S61653). Upon collection, explanted lungs were cannulated, inflated to 30 cm water pressure, and frozen over liquid nitrogen vapors at 10 cm H2O pressure, then stored at −80°C. Regions of the lung with obvious pathology (ie, oedema, inflammation, or infection) were excluded based on inspection of gross lung slices and the CT screening.

Cores were divided into several pieces for nuclei isolation, histology, and other protocols outside those included in this study. For nuclear isolation, we applied a modified version of the nuclei extraction using Optiprep protocol (https://www.protocols.io/view/frankenstein-protocol-for-nuclei-isolation-from-f-bqxymxpw). Briefly, samples were homogenized in Nuclei EZ lysis buffer (Sigma-Aldritch) with MACS C-Tubes using a gentle homogenization setting. Homogenate was filtered using 100 µm strainer, then centrifuged at 500G for 5 min. Nuclear pellet was then purified by mixing with 300 µL G30 cushion solution buffer (50:50 mix of 60% Optiprep (Iodixanol) and nuclei wash buffer) and underlaying with 1 mL G30 buffer, then centrifuged at 500g for 5 min at 4ºC. Remove supernatant and wash pellet with 1 mL nuclei wash buffer, centrifuge and resuspend in 300 µL nuclei resuspension buffer. Isolated nuclei were counted using the Countess cytometer then processed according to 10x 3-prime v2.1 single cell sequencing protocols (10x Genomics). Sequencing of barcoded libraries was done using the Illumina HiSeq4000 for paired-end reads.

**Data Integration**

The calculation of the correction vectors does not need to be performed in the original dimensions. Instead, a subset of genes such as highly variable genes (HVGs) is sufficient for computing the correction vectors. We used the intersection of highly variable genes (HVGs, using the sc.pp.highly_variable_gene() function in scanpy) between the reference and query dataset to compute the correction vector. IPF and Carraro control data, were treated as separate query batches and HLCA was used as the only reference for data integration.

**Evaluation Metric**

We used R2 score to evaluate the model performance in LOO and CD test, which is defined by the following equation:

$$R^{2}=1-\sum_{i=1}^{m} \frac{{(y_{i}-\hat{y_{i}})}^{2}}{{(y_{i}-\bar{y})}^{2}}$$

Where $y_{i}$and $\hat{y_{i}}$ denotes the true donor age and predicted donor age for the ith donor, and $\bar{y}$ denotes the mean of these true values.

**Calculation of Feature Importance and P-values**

We used SHAP values to compute the importance of the genes selected by the model. The SHAP value represents how much impact a change in the input can have on the output compared to using a set of reference examples as inputs. In this study, we defined a reference example as an input vector with all entries equal to zeros. We used all available donors in the HLCA dataset to compute SHAP score vectors. Each vector gives the importance of each input feature for a specific donor. The absolute values of SHAP score vectors across all donors were summarized as final scores for features. The importance of each gene is the sum of SHAP scores from all of its polynomial features (first/second degree of expression mean and first/second degree of expression variance). For PCA transformed features, we performed the following computation to obtain gene-wise importance:

$$s^{'}=\boldsymbol{A}^{+}s$$

Where $s$ is the SHAP score vector, $s\in\mathbb{R}^{k}$, aggregated as the sum of absolute SHAP values from all donors and all polynomial features computed from the same PC were also aggregated together in this vector. $k$ is the number of top PCs ($k$ = 10). $\boldsymbol{A}^{+}$ denotes the Moore-Penrose pseudo-inverse of the eigen vector matrix from PCA, $\boldsymbol{A}\in\mathbb{R}^{k\times g}$ and $\boldsymbol{A}^{+}\in\mathbb{R}^{g\times k}$. $g$ is the number of marker genes. This operation can transform the PCA based SHAP scores back to the original gene dimensions ($s^{'}$).

We computed p-value for each gene using a permutation-based approach. We randomly reshuffled the expressions of one particular gene among all cells and performed feature extraction computation, model training, and SHAP score computation as described above. Each round of reshuffling would generate a SHAP score under random perturbation. We then repeated this process for 100 times to generate a null distribution of SHAP score for one gene. The assumption of this reshuffling process is that a null distribution of SHAP scores only represents the importance of a random feature. The p-value is then calculated as number of times the given SHAP score is smaller than the empirical null distribution of SHAP scores.

**RESULTS**

**Including Variance Features in the Model**

We also explored whether using variance features, which is unique to PolyEN, improves model performance for the top cell types as presented in Fig. 3 and Fig. 4. We found that variance features indeed resulted in a consistent improvement in both smoker and non-smoker group (S6 Fig). Finally, we tested different polynomial degrees and found that degree 2 polynomials led to a better performance in smoker group but showed a slightly worse performance when compared to degree 1 polynomials for non-smokers.

**Temporal Expression Patterns of Polynomial Features**

Additionally, we selected the three basal-related cell types (basal, basal resting, and suprabasal) and explored how the union of senescence markers compare to all expressed genes for these cell types. Shown in S8 Fig are the temporal patterns of genes with significant SHAP scores (see Materials and Methods) over different ages. Similar to the pattern of *PMAIP1* presented in Fig. 5B, we can observe a distinct two-module pattern for basal, basal resting and suprabasal cells in both significant genes of senescence markers and significant genes of all expressed genes. We further explored whether the top ranked genes differ between these two types of gene lists. The senescence markers yielded 67, 63, and 36 significant genes for basal resting, basal, and suprabasal cells respectively while all expressed genes yielded 181, 35, and 202 significant genes for basal resting, basal, and suprabasal cells respectively. For senescence markers, there are 14 common significant genes among the three cell types (p-value = 0.0, see MATERIALS AND METHODS and Fig. 5C). For all expressed genes, there are 7 common significant genes among the three cell types (p-value = 0.0, see MATERIALS AND METHODS and Fig. 5C). We found senescence markers for the three basal-related cell types tend to consistently rank general transcription factors such as *FOS* and *JUN* at the top while all expressed genes captured genes with more specific roles (*THOC3* and *PMAIP1*/NOXA, Fig. 5C) at the top. Specifically, *FOS* and *JUN* are known for their ability to form transcription factor complex AP-1, which is broadly involved in cell proliferation (1), differentiation (2), transformation (3), and apoptotic cell death (1). *THOC3* is a component of transcription export complex (TREX) that assists mRNA export into cytoplasm (4) and *PMAIP1*, as discussed previously, is a known apoptosis activator. This difference may be explained by the fact that 1) all expressed genes captured apoptotic related genes not collected in the senescence marker lists and 2) all expressed genes captured genes in the pathways related to aging process but not directly related to cellular senescence.

**DISCUSSIONS**

Age prediction from transcriptomic signatures is a long-standing challenge. In addition to the methods we benchmarked in this study, several other methods have also been developed in recent years to address this task. Peters et al modeled aging in human peripheral blood cells by using a ridge regression model to capture gaussian random effect of gene expressions (5). Jian et al modeled chronological age of human CD8^+^ T cells using mixed-effect elastic net model and mixed-effect random forest model (6). BURNS is an online age-prediction tool based on a plethora of machine learning models trained across multiple human tissues (7). For species other than humans, BiT age is a tool developed based on trained elastic net model for predicting age of C. elegans (8). However, these methods either did not provide code for model training/testing or the documentation for applying the pipeline to new data is incomplete.

Notably, our analysis reveals differences in aging signatures in the transcriptomes of smokers and nonsmokers. Differences in both the cell type and gene level contributions to the model were observed, with epithelial cell signatures primarily contributing for nonsmokers and endothelial/mesenchymal signatures for smokers. Moreover, the union of senescence markers outperformed the set of all expressed genes in the case of smokers. This suggests that there are potentially differences in the regulation of cellular senescence with aging in smokers.

While the work here utilized single cell data, prior work using bulk data has also identified similar cell types, though at a less refined level (9). In that work, gene co-expression networks were developed using bulk RNAseq. The network most strongly associated with aging was expressed in type II alveolar epithelial cells and the network most enriched for cellular senescence was primarily expressed in endothelial cells. Here, we further refined these findings but identifying both, cell types and specific gene combinations that can be used to predict age from lung transcriptomics data.

**REFERENCES**

1. Wu,Z., Nicoll,M. and Ingham,R.J. (2021) AP-1 family transcription factors: a diverse family of proteins that regulate varied cellular activities in classical hodgkin lymphoma and ALK+ ALCL. *Exp. Hematol. Oncol.*, 10.1186/s40164-020-00197-9.

2. Eckert,R.L., Adhikary,G., Young,C.A., Jans,R., Crish,J.F., Xu,W. and Rorke,E.A. (2013) AP1 Transcription Factors in Epidermal Differentiation and Skin Cancer. *J. Skin Cancer*, 10.1155/2013/537028.

3. Shaulian,E. and Karin,M. (2002) AP-1 as a regulator of cell life and death. *Nat. Cell Biol.*, 10.1038/ncb0502-e131.

4. Cheng,H., Dufu,K., Lee,C.S., Hsu,J.L., Dias,A. and Reed,R. (2006) Human mRNA Export Machinery Recruited to the 5′ End of mRNA. *Cell*, 10.1016/j.cell.2006.10.044.

5. Peters,M.J., Joehanes,R., Pilling,L.C., Schurmann,C., Conneely,K.N., Powell,J., Reinmaa,E., Sutphin,G.L., Zhernakova,A., Schramm,K., *et al.* (2015) The transcriptional landscape of age in human peripheral blood. *Nat. Commun.*, 10.1038/ncomms9570.

6. Lu,J., Ahmad,R., Nguyen,T., Cifello,J., Hemani,H., Li,J., Chen,J., Li,S., Wang,J., Achour,A., *et al.* (2022) Heterogeneity and transcriptome changes of human CD8+ T cells across nine decades of life. *Nat. Commun.*, 10.1038/s41467-022-32869-x.

7. Shokhirev,M.N. and Johnson,A.A. (2021) Modeling the human aging transcriptome across tissues, health status, and sex. *Aging Cell*, 10.1111/acel.13280.

8. Meyer,D.H. and Schumacher,B. (2021) BiT age: A transcriptome-based aging clock near the theoretical limit of accuracy. *Aging Cell*, 10.1111/acel.13320.

9. Man,R. De, McDonough,J.E., Adams,T.S., Manning,E.P., Myers,G., Vos,R., Ceulemans,L., Dupont,L., Vanaudenaerde,B.M., Wuyts,W.A., *et al.* (2023) A Multi-omic Analysis of the Human Lung Reveals Distinct Cell Specific Aging and Senescence Molecular Programs. *bioRxiv*, 10.1101/2023.04.19.536722.

**
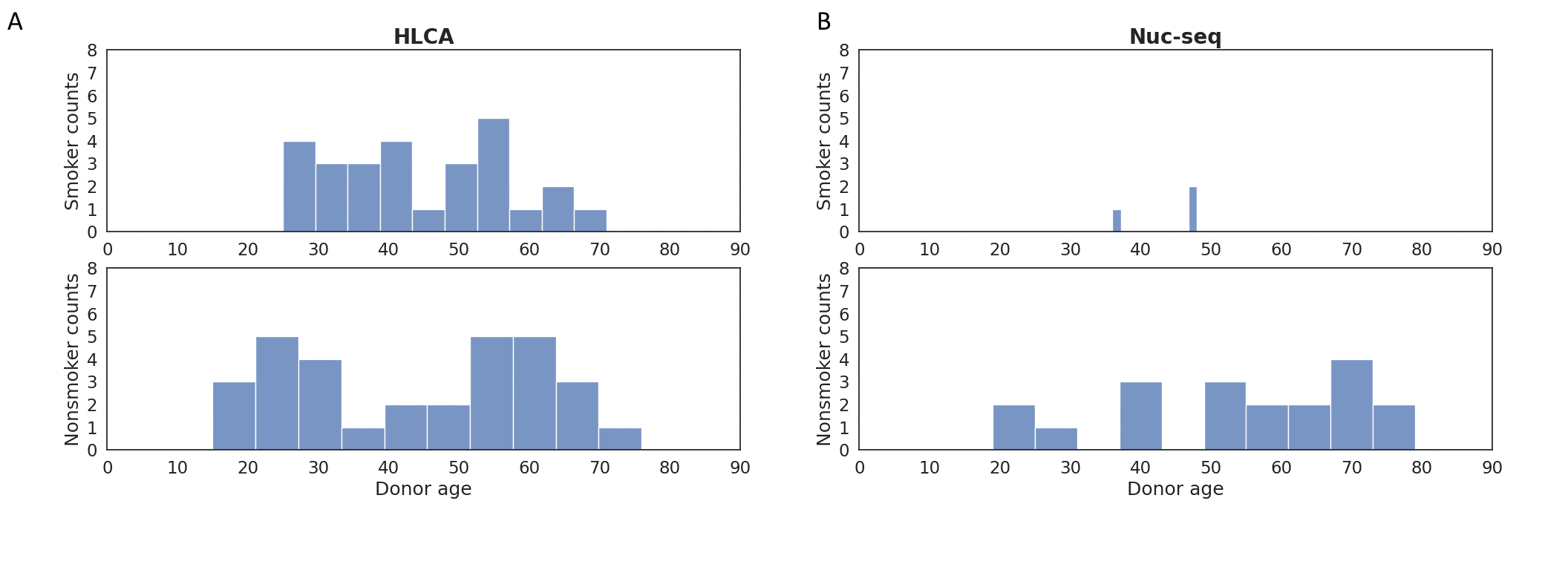
**

**Fig A. Donor age distribution of the different datasets.**


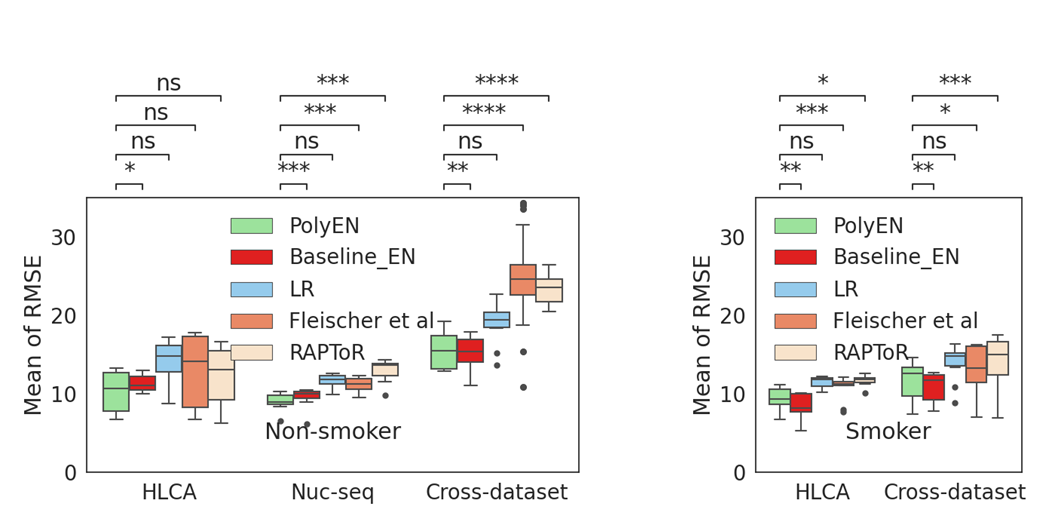


**Fig B. RMSE for the same evaluation performed in fig. 2c.** The same evaluation as fig. 2c is performed here. Left: results from non-smokers; Right: results from smokers.

**
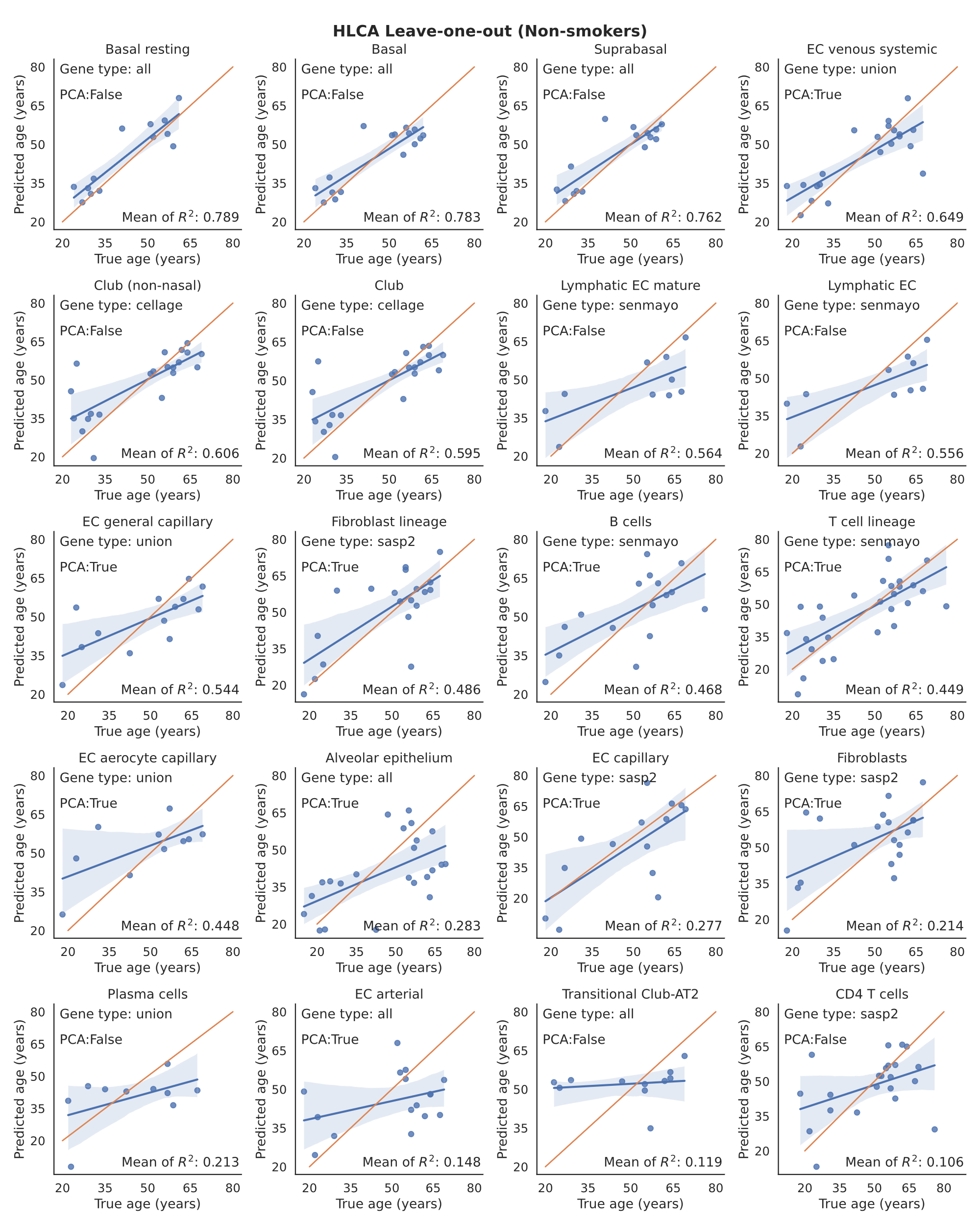
**

**Fig C. Predicted donor ages VS true donor ages for LOO test on non-smoker group of HLCA.**

**
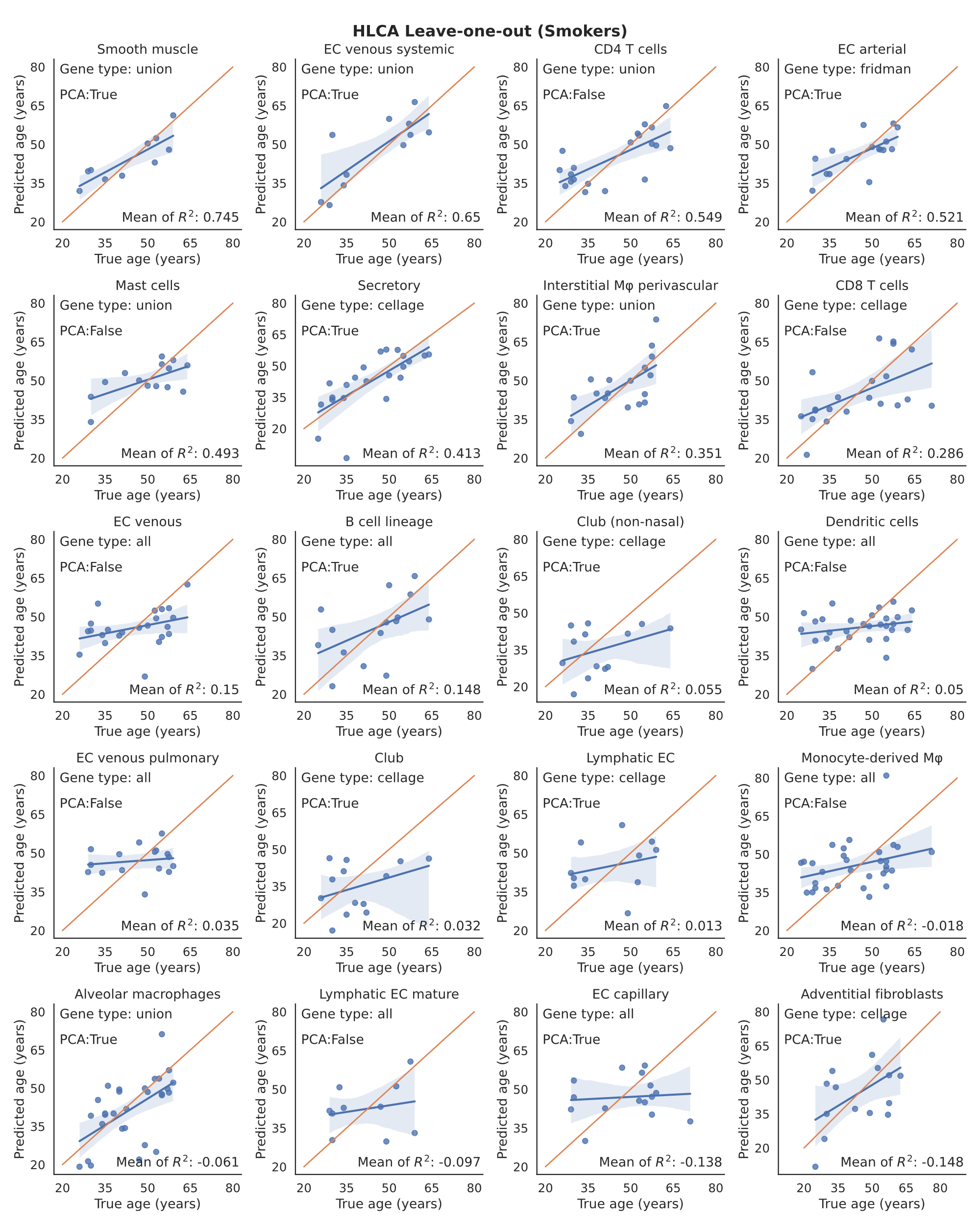
**

**Fig D. Predicted donor ages VS true donor ages for LOO test on smoker group of HLCA.**

**
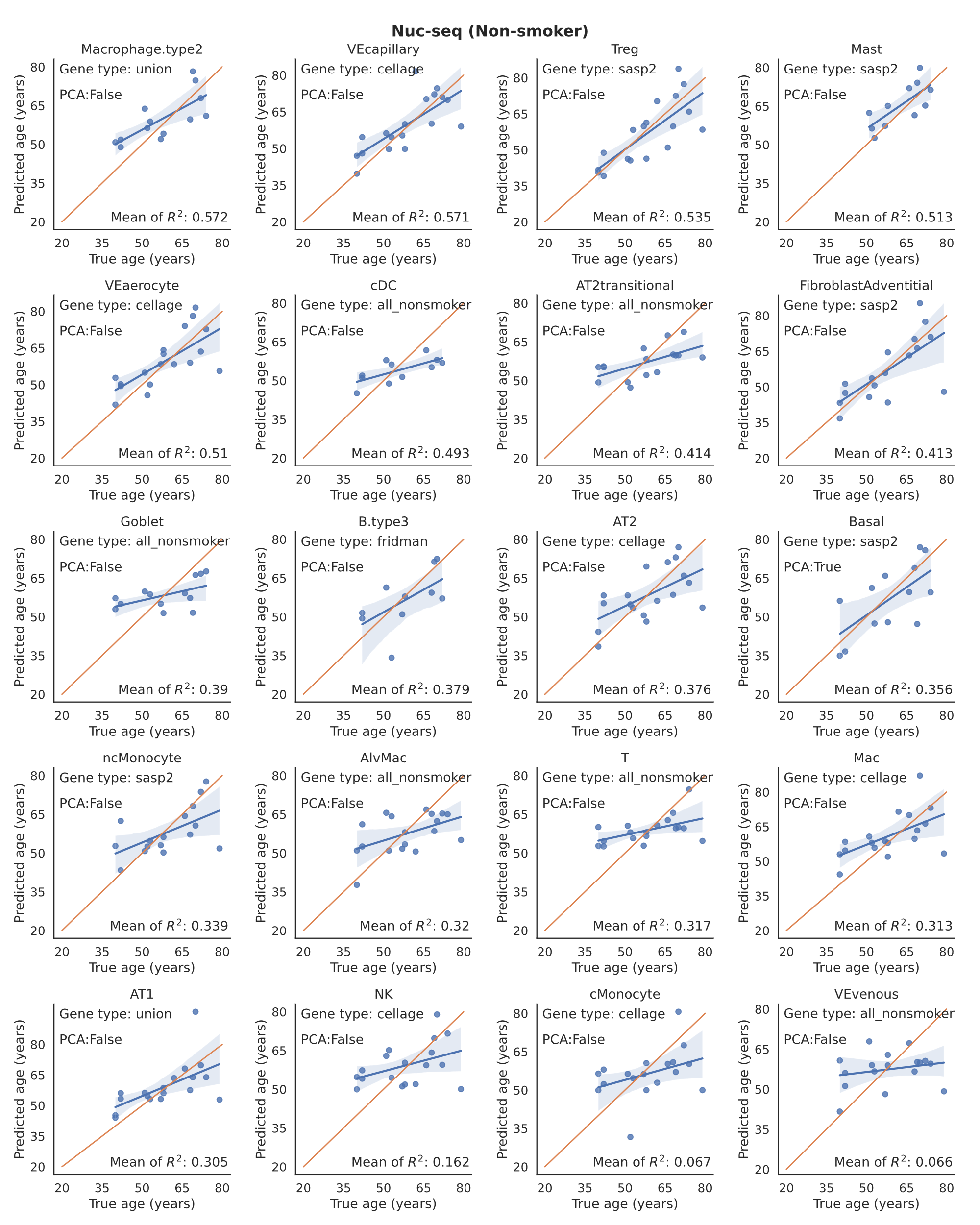
**

**Fig E. Predicted donor ages VS true donor ages for LOO test on smoker group of nuc-seq.**

**
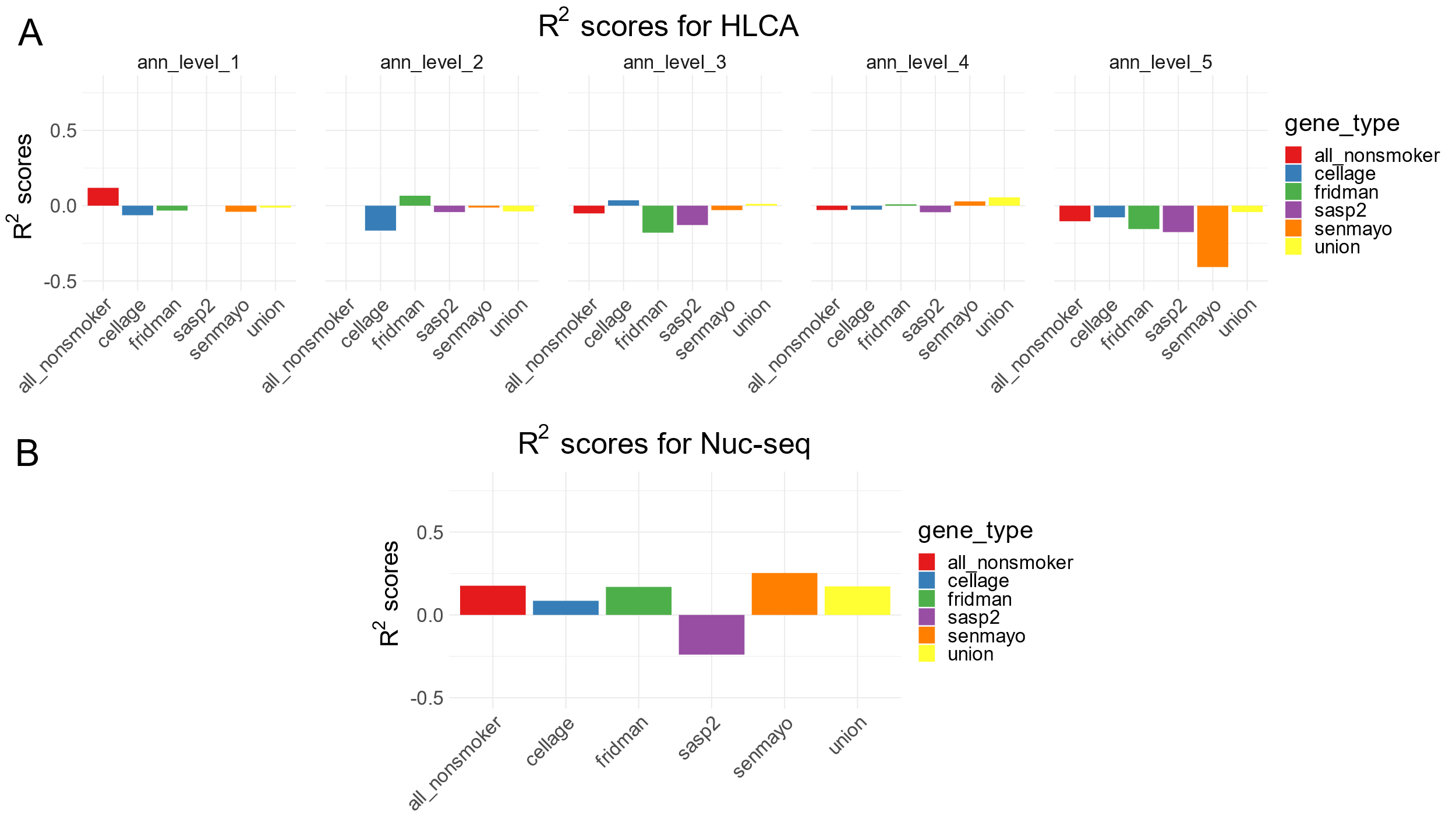
**

**Fig F. Performance of the meta regressor for the HLCA and Nuclear-seq dataset.**

**
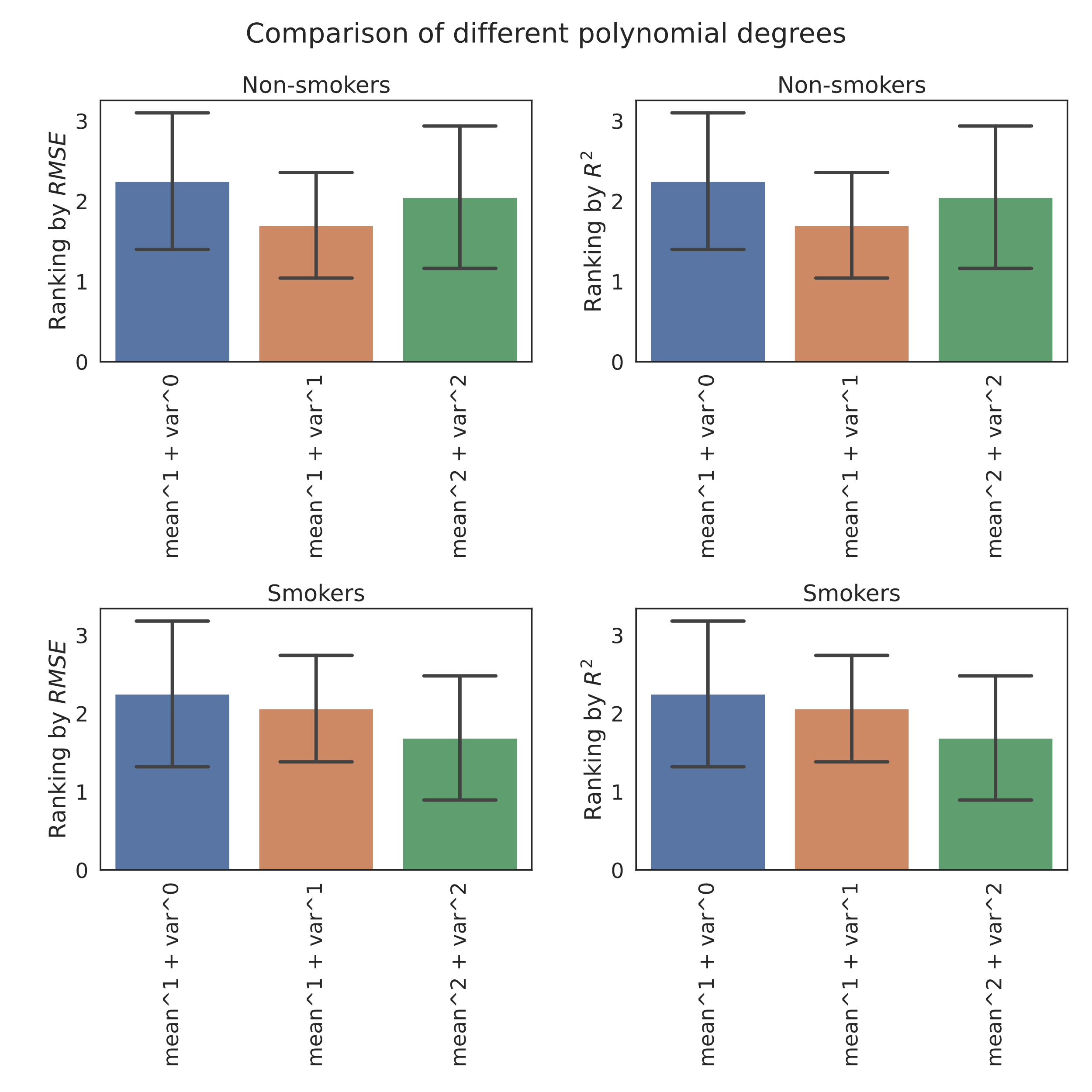
**

**Fig G. Comparison of different polynomial degrees.** Each plot shows the relative ranking among the three types of polynomial settings shown on X axis. We ranked the polynomial types within each row combination (cell type + gene type + PCA type) shown in fig. 3 and fig. 4. **mean^1 + var^0**: first degree of expression means and no variance features included; **mean^1 + var^1**: first degree of expression means and first degree of expression variances included; **mean^2 + var^2**: first and second degree of expression means and first and second degree of expression variances included.

**
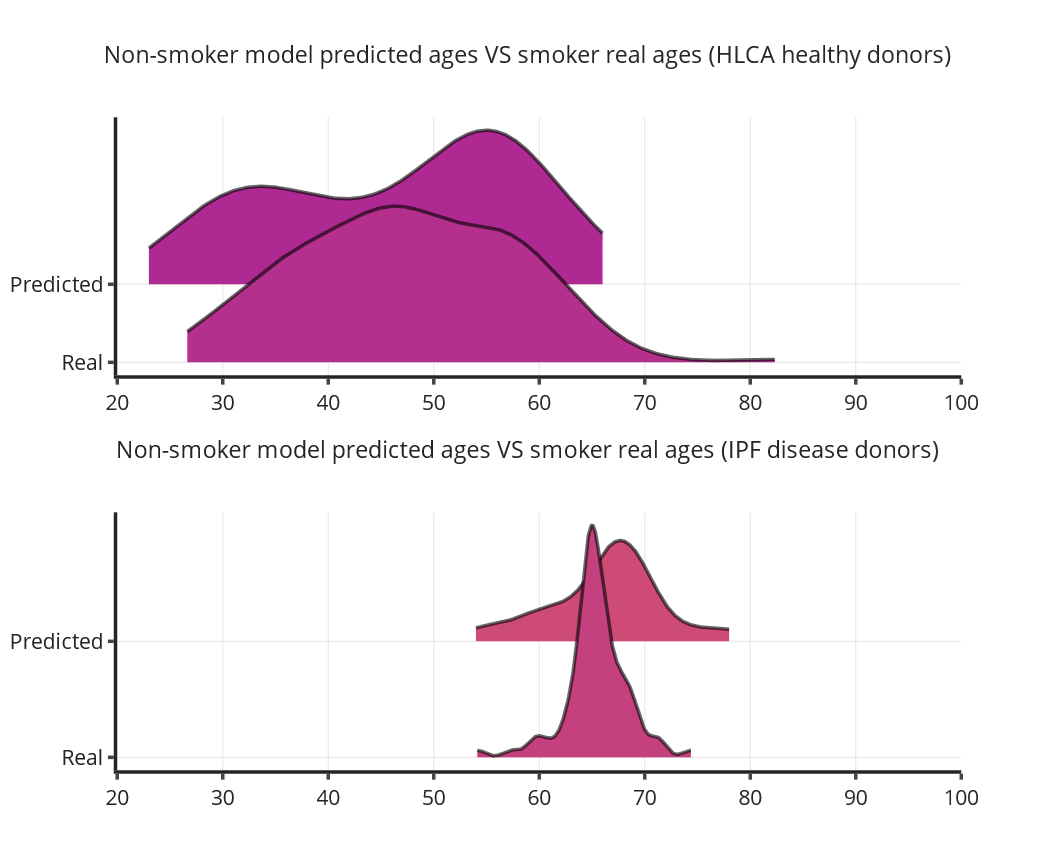
**

**Fig H. The distribution for predicted ages and real ages when non-smoker models were used to predict smoker age.**


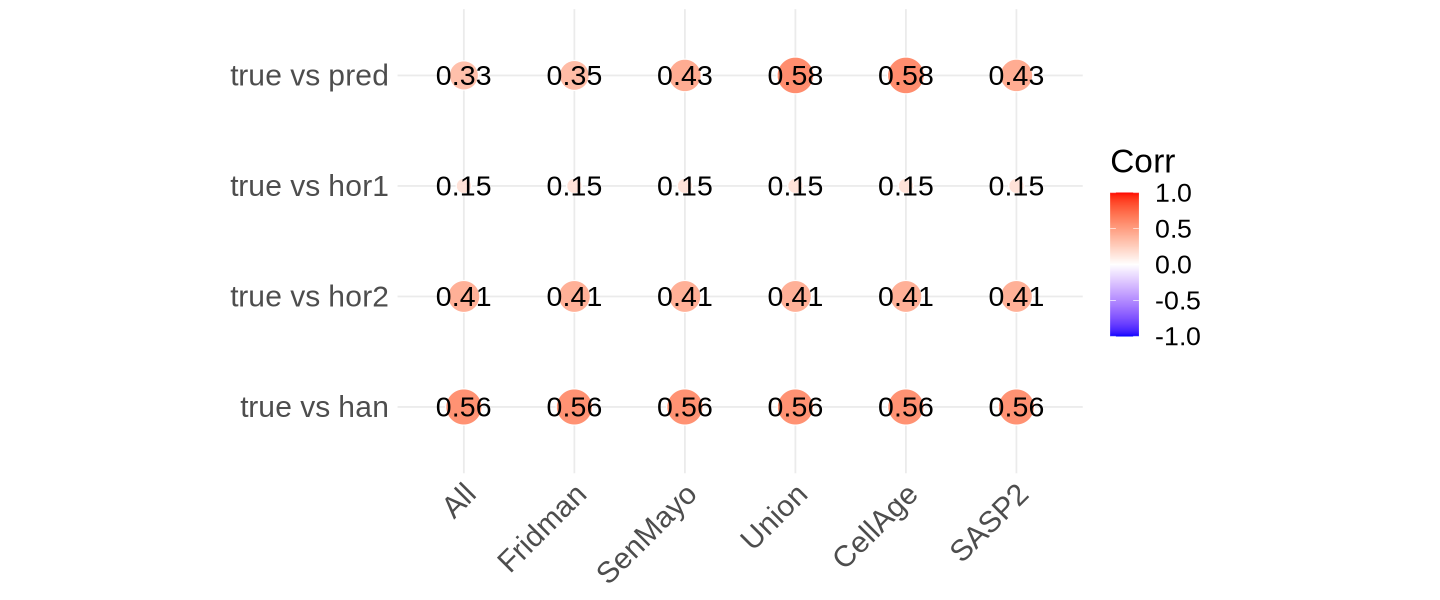


**Fig I. Comparison between transcriptomic ages and methylation ages.** Gene expressions were averaged across all available cells in the Nuclear-Seq dataset and models were trained and ages were predicted using the same steps as in Fig. 5B. Numbers shown in the plot stand for Pearson correlation coefficients. “true” represents true chronological donor age and “pred” represents transcriptomic ages predicted by polyEN. “hor1” is methylation ages predicted by Horvath1 method; “hor2” is methylation ages predicted by Horvath2 method and “han” is methylation ages predicted by Hannum method.

**
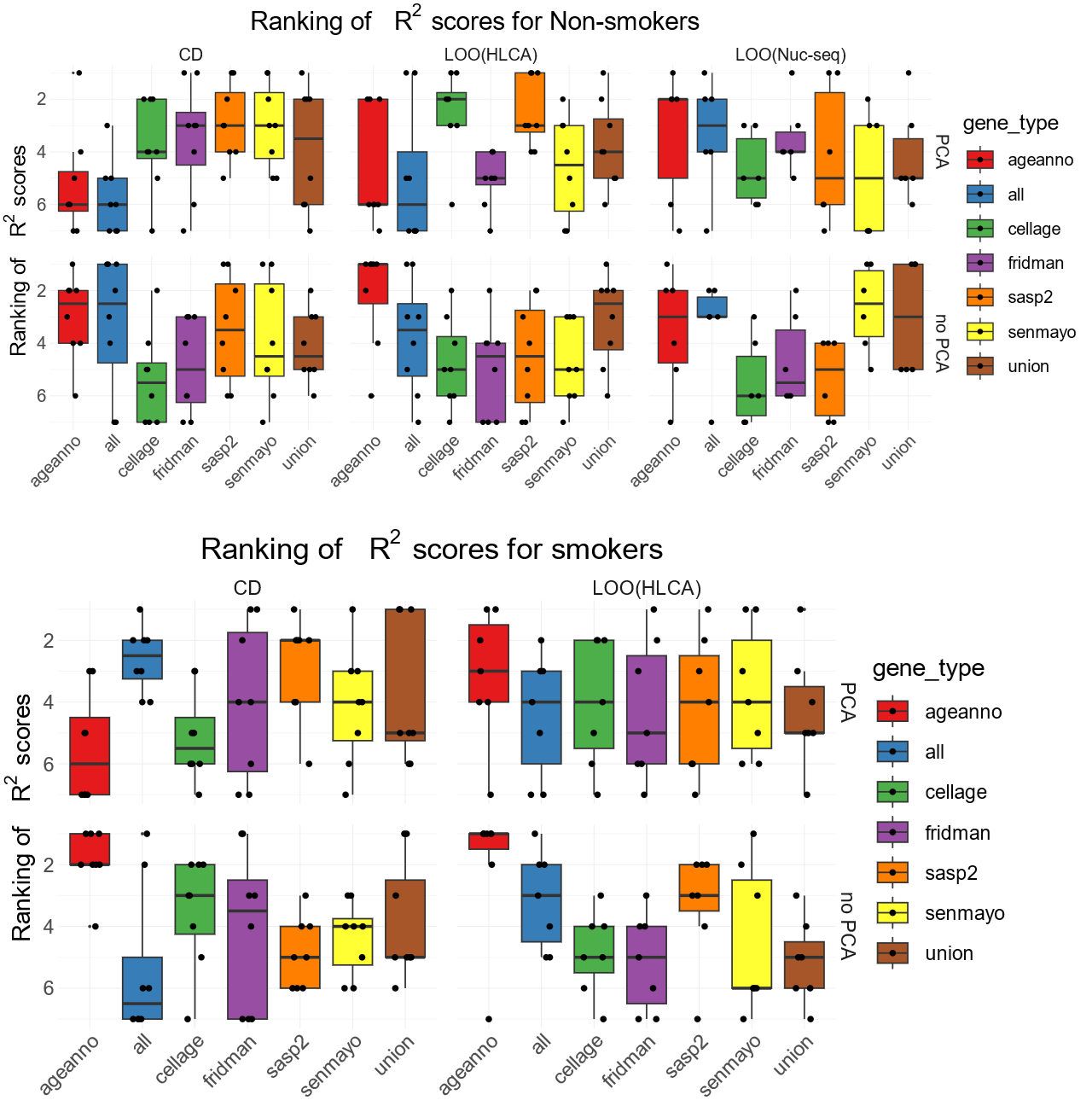
**

**Fig J. Evaluation of senescence marker lists and AgeAnno aging makers.** Rankings of R^2^  scores were computed for the cell types available in AgeAnno database (alveolar cell, B cell, endothelial lymphatic cell, epithelial cell, fibroblasts cell, mast cell, myeloid cell and T cell).

**
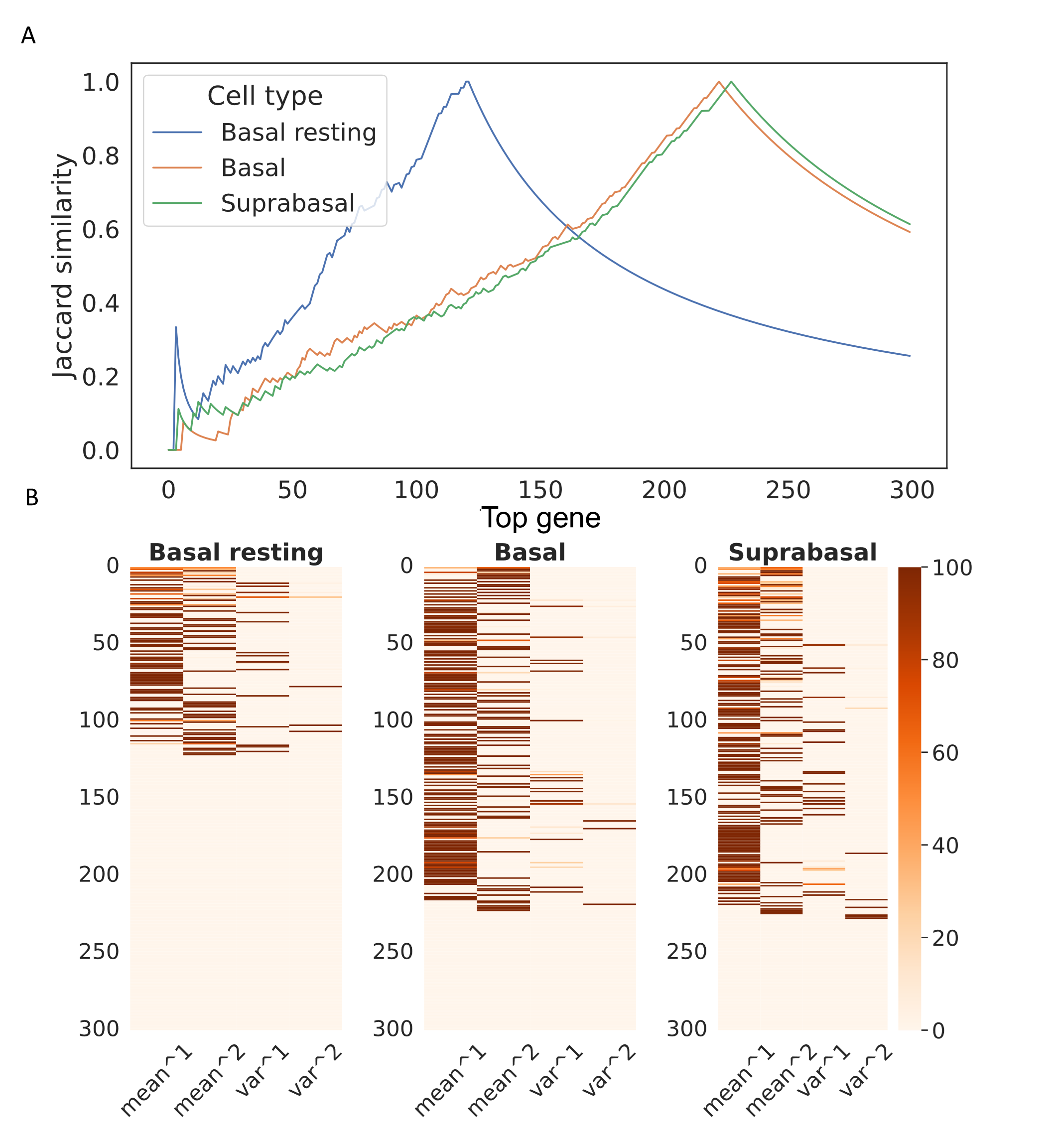
**

**Fig K. Coefficients VS SHAP-based importance scores and first-order VS second order polynomial features. A.** The comparison between the two top-ranked 300 gene lists generated respectively by coefficients from PolyEN model and SHAP-based importance scores. Jaccard similarity score was used to compute the similarity between the two lists when genes are iteratively included from top1 to topN (1 ≤ N ≤ 300). The drop of similarity is a result of zero importance scores after the tipping point. SHAP-based importance scores will generate arbitrary rankings after the tipping point and therefore comparing these genes would be meaningless. **B.** The relative weights of first-order, second-order features. For each gene we computed the SHAP-based importance scores respectively for the four features (first-order, second-order expression means, and first-order, second order expression variances). Then the weight of each feature is the importance score of each feature divided by the sum of importance scores from all four features. Weights are visualized as percentages.


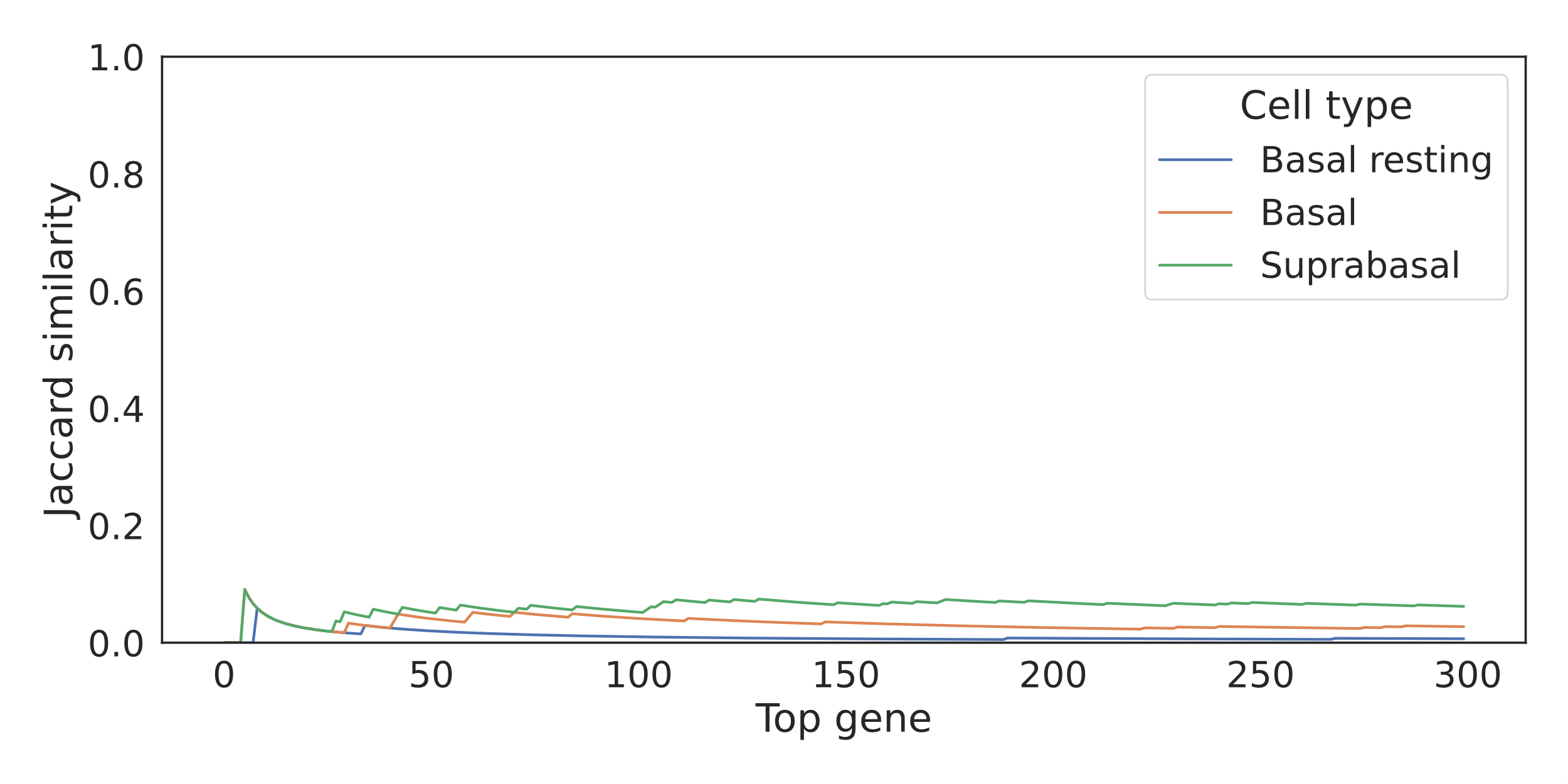


**Fig L. SHAP-based importance scores VS top-ranked highly expressed genes.** 300 top genes as ranked by SHAP-based importance scores were compared to 300 top genes ranked by expressions. Jaccard similarity score was used to compute the similarity between the two lists when genes are iteratively included from top1 to topN (1 ≤ N ≤ 300).

**
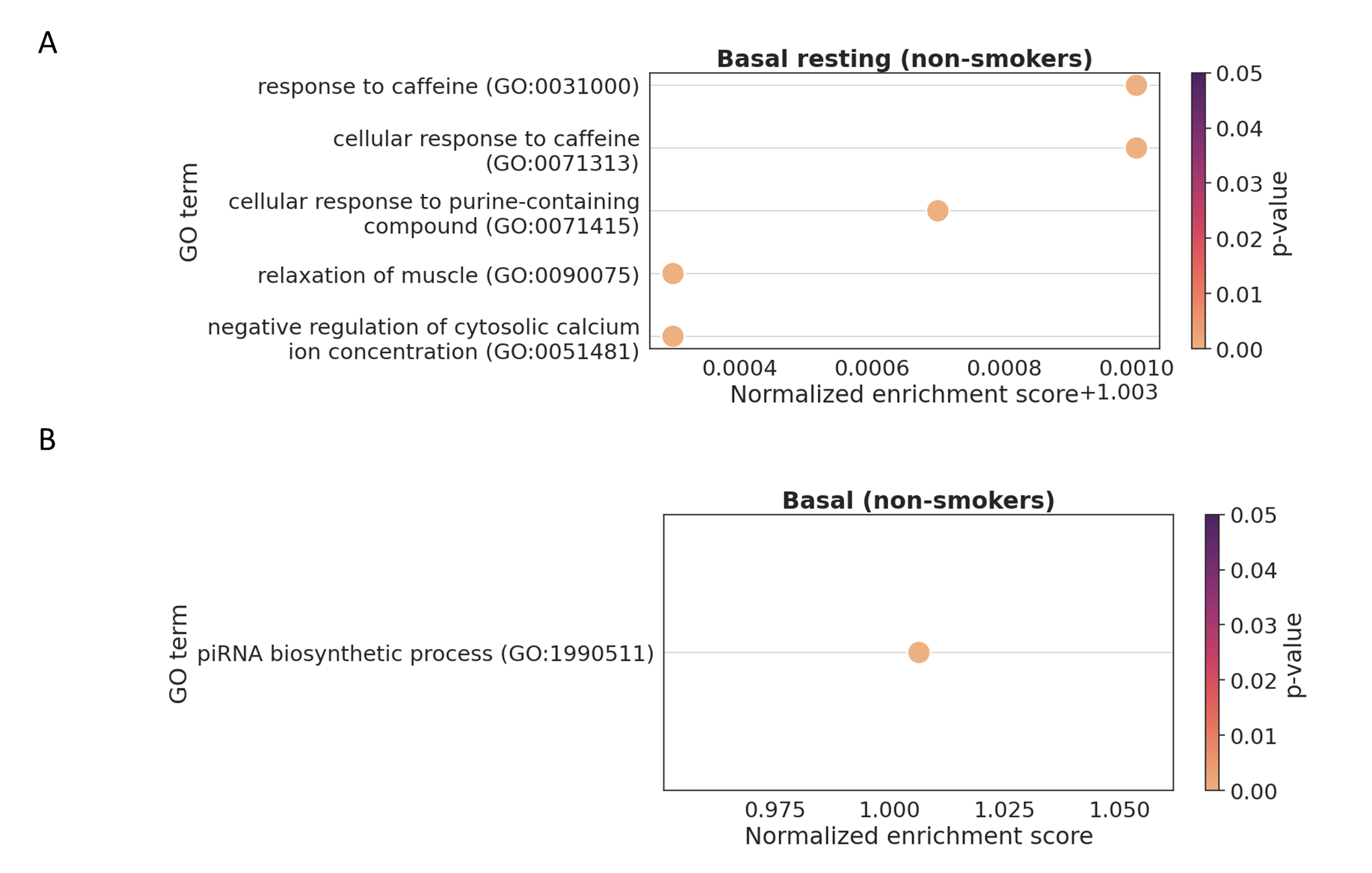
Fig M. GSEA results for coefficients-based gene ranking.** Similar to Fig. 5B, GSEA was performed to coefficients-based gene ranking. The top five significant GO terms for basal and basal resting cells from the nonsmoker group were shown here. No enriched terms found for suprabasal cells.


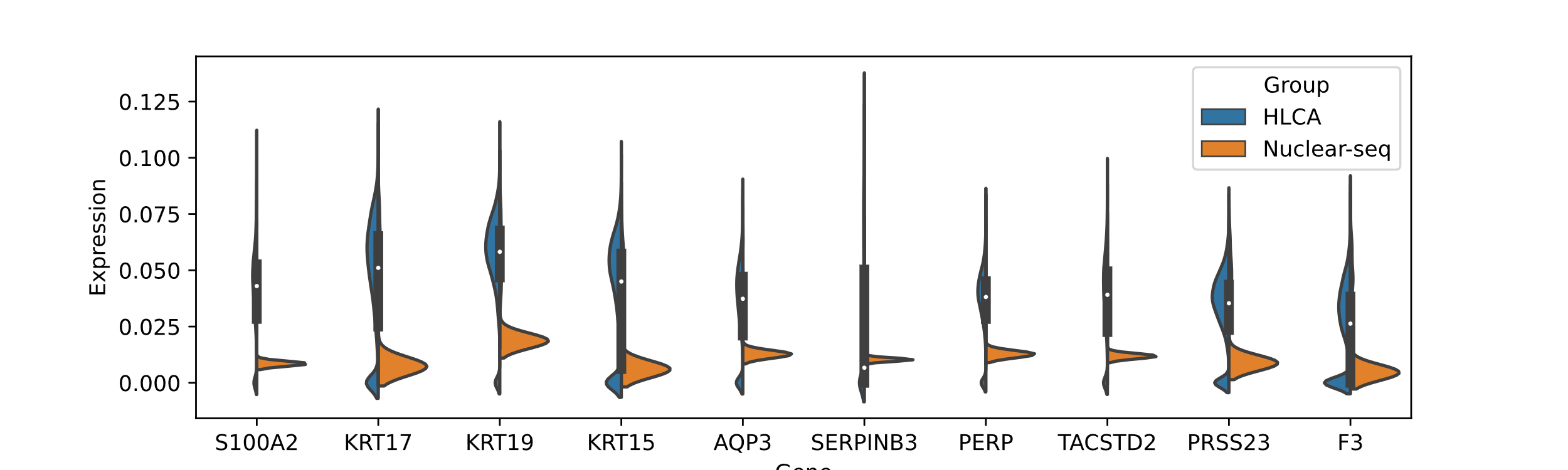


**Figure N. Comparison of marker gene expressions for basal cells in the HLCA dataset and the Nuclear-seq dataset.** We used mnnpy integrated Nuclear-seq dataset and the original HLCA dataset to generate the plot. We first identified the marker genes for basal cells by comparing the expressions of basal cells to all other cells in the HLCA dataset using the ‘logreg’ method included in the function ‘sc.tl.rank_genes_groups()’ of the Scanpy package. The top 10 gene markers were selected based on highest scores. We then examined top 10 marker expressions of basal cells in HLCA and Nuclear-seq dataset.
