# Supplementary material for: Predicting lung aging using scRNA-Seq data
Source: PLoS Comput Biol. 2024 Dec 19;20(12):e1012632. doi: 10.1371/journal.pcbi.1012632 (PMC11741621; doi:10.1371/journal.pcbi.1012632)
Supplement: Attachment — Submitted filename: polyEN_response_letter_rev1_08-16-2024.docx [file pcbi.1012632.s007.docx]

*We wish to thank the reviewers for their constructive feedback and critical comments. We provide our point-by-pint response below.*

**Reviewer #1:**

1. How to predict age for each cell type of lung from the methylation data is not clear. Did authors use single cell DNA methylation? A description of the methylation data used here is needed. Does the methylation data is profiled from the same cohort of individuals with the single cell RNA-seq data? I guess the three methods (Horvath, Hannum, and GrimAge) were trained on the bulk methylation data. If authors used single cell methylation data as input, how does it affect the results? The polyEN results are trained and tested on the similar datasets, however this is not the case for the methylation-based predictions.

*We thank the reviewer for this comment. We have now clarified the type of methylation data we used. Specifically, we used bulk Methylation data. These were profiled using the Infinium Human Methylation 450K Bead Chip. Samples are taken from the same donors from which we obtained the Nuclear-Seq data. Whole methylome data was performed on one slice of each sample. Therefore, PolyEN-based age predictions and methylation-based aging clocks calculations were performed on the same set of donors to ensure a fair comparison. We now include this information in section* ***Methylation data processing*** *which is part of* ***MATERIALS AND METHODS****.*

**Reviewer #2:**

Major comments:
The idea of modeling the features as first- and second-degree polynomials is interesting, and the intuition behind it makes sense since the quadratic form could capture feature trends beyond the linear formulation. Investigating age regression performance that is consistent for certain cell types and gene sets helps understand aging in lungs among smokers and non-smokers. However, it would be helpful to include more detailed descriptions of the methodological setup and modeling choices to robustly support the results of the paper (see points below).

First, what was the intuition behind calculating means and variances instead of using single-cell gene expression read-out directly (as done in previous single-cell age prediction studies)?

*Since age is a function of an individual and not a function of cell, any method for predicting aging needs to combine information from all cells (or at least all cells of the same type for cell-type-specific predictions). Prior methods have also aggerated this information, usually using only the average without considering the temporal aspects. Our method is different in two significant ways. First, we additionally included the expression variances since previous studies have shown that older individuals have more variable gene expressions, even in the same cell types (Bahar et al. 2006; Enge et al. 2017; Martinez-Jimenez et al. 2017). Second, we used polynomials to combine measurements across time. As the reviewer mentions, such modeling allows for ‘smoothing’ of the individual time points (ages) which helps in reducing overfitting, especially when dealing with heterogenous data from several different individuals. We now include the discussion in Model training and testing section under MATERIALS AND METHODS.*

For any normalization and dimensionality reduction done in the paper, was it ensured that it was done after the training/test split to avoid data leakage? Also, was a similar hyperparameter tuning scheme used to pick the best hyperparameters for the baseline models as well? What was the hyperparameter grid that was explored for them?

*Since our data was based on individuals (and not cells), there is no data leakage. Specifically, for normalization, we used CPM normalization which normalizes each cell by its library size so normalization for each individual was performed independently. For dimensionality reduction, we indeed isolated the training and testing process. We performed dimensionality reduction on the training set only and then used the PCA loadings learned from the training set to perform dimensionality reduction on the test set. Since the PCA step is unsupervised and does not use any information on the age of the individual, there is no leakage between the cohorts. The hyperparameters tunning were also performed only on training dataset and then applied to testing set. Instead of grid search, we used python package Hyperopt to perform the optimal hyperparameter search. Hyperopt performs a sequential model-based optimization which sequentially approximates the optimal hyperparameter set by a surrogate model. We set maximum number of evaluations as 30 for Hyperopt based tunning. You may find details in* ***Model training and hyperparameter tunning*** *section of* ***MATERIALS AND METHODS****. The hyperparameters for our methods were sampled in the following spaces: α was searched within the set {0.001, 0.01, 0.1, 1, 10, 100} and ρ is searched within the continuous interval [0.1,1.0] (This information can be found in the section* ***Model training and testing*** *in* ***MATERIALS AND METHODS****). For other methods we compared to, we did the following. For LR with L1 regularization we searched for the best hyperparameters for the regularization term α in the same search space and using the same approach as we did for PolyEN. For the other two methods, we followed the guidelines in their GitHub pages to run the methods. We have clarified this in the section* ***Comparison to other methods*** *under* ***MATERIALS AND METHODS.***

It is unclear why simple linear regression was chosen as a baseline when one could pick an elastic net with various ablations on the feature types as a baseline to better support the final formulation of the PolyEN model.

*As the reviewer suggested, we have now added a baseline elastic net models to the set of methods we are comparing against. Results, presented in fig. S7 show that PolyEN improves on elastic net using the base expression features (tested on HLCA dataset by leave-one-out test). The baseline elastic net model is equivalent to an elastic net model trained with only first-degree expression means (mean^1 + var^0 in Fig. S7). We ranked the R2 scores and RMSE scores obtained from testing different polynomial types within each row (cell type + gene type + PCA type) shown in fig. 3 and fig. 4. We sorted RMSE scores by ascending order and R2 scores by descending order. This ensures that lower rankings in the plot always indicate better performances. Specifically, the average RMSE score rankings of the baseline elastic model are both 2.25 for non-smokers and smokers, which are worse than rankings from other settings, suggesting worse performances using the baseline elastic net model (Fig. S7). Similar observation can be seen for average R2 score rankings, which is 2.25 for both non-smokers and smokers and is worse than rankings from other settings (Fig. S7).*

The scales in Figure 2C make it very hard to compare the boxplot performance. The cross-dataset results do not seem very good, raising questions about the model's generalizability.

*We thank the reviewer for raising this point. We agree that the results are not great, though* *it is worth noting that polyEN is much better than other methods, at least for the non-smoker data (Fig. 2C). We believe this was due to relatively small sample sizes for each cell type (See supplementary file3). When we further split the HLCA data into training and test sets, there are only limited donors available for each cell type in both training and testing sets, making cross-dataset evaluations very challenging. With more samples and further developments in methods, we may be able to see improved performance for cross-dataset benchmark. We now include this information in* ***Discussion****.*

It would be interesting to investigate important features obtained via the coefficients of the elastic net model. Do important genes overlap with the SHAP genes? I am also curious to learn how the paper is interpreting a SHAP importance score for a first-order versus second-order polynomial feature? Does it connect to the intuition behind using this formulation?

*We thank the reviewer for the suggestion. To compare the use of coefficients vs. SHAP values, we performed the following analyses. We aggregated the absolute values of coefficients of the polynomial features for each gene. We then ranked the genes by the aggregated absolute coefficients. We compared the resulting rankings to the rankings generated by SHAP-based importance scores. This generated two gene ranking lists.* *Jaccard similarity score was used to compute the similarity between the two lists when genes are iteratively included from top1 to topN (1 ≤ N ≤ 300). The results shown in Fig. S10A indicate higher level of disparity for top-ranked genes between the two lists and more overlapped genes when expanding the gene lists to lower-ranked genes in the top 300. This observation highlights the uniqueness of the top-ranked genes selected by SHAP-based importance scores.*

*As for first- versus second-order degree features, we followed the steps below to examine their difference. To address this comment, for each gene we computed the SHAP-based importance scores respectively for the four features (first-order, second-order expression means, and first-order, second order expression variances). We then used the same top 300 genes in Fig. S10A and calculated the weight of each type of feature in each gene.* *The results as visualized in Fig. S10B show that for top-ranked genes, both types of features (first and second order) are needed. Many top-ranked genes show distinctive differences between the first-order and second-order features, suggesting that using higher-order features can capture additional knowledge about age related genes.*

I could not read the text in Figure 3, which made it harder to understand the comparison of transcriptomics and methylation results.

*Thank you for your suggestion. We have remade Fig. 3 and Fig. 4 with larger fonts and moved some text into supplementary file 3.*

Minor points:

A proof-reading pass of the paper (and the abstract) is highly recommended

*Thank you for your feedback. We have revised the manuscript and corrected typos accordingly.*

Higher-resolution figures with bigger font sizes are highly recommended.

*Thank you for your comment. We have remade all figures in the main text with bigger font sizes.*

CellBiAge was not included in the baseline comparison as it performed classification, but would the feature binarization step help the predictive performance on this dataset?

*Age classification may be tricky since it requires hyperparameters to set the bins. Continuous age prediction is more natural and less arbitrary. Thus, we did not compare directly to CellBiAge. As for binarization of expressions, we believe this may eliminate or diminish the cell-cell variability, which is an important feature in elder individuals (Bahar et al. 2006; Enge et al. 2017; Martinez-Jimenez et al. 2017).*

The main finding of the temporal analysis should be included in the main manuscript if it is mentioned. Also, its description in the Supplementary was difficult to follow.

*Thank you. As suggested, we have moved the temporal analysis (originally in supplementary file, fig. S10) results into main text (now Fig. 6) and revised the description accordingly.*

*References*

*Bahar R, Hartmann CH, Rodriguez KA, Denny AD, Busuttil RA, Dollé MET, Calder RB, Chisholm GB, Pollock BH, Klein CA, et al. 2006. Increased cell-to-cell variation in gene expression in ageing mouse heart. Nature.*

*Enge M, Arda HE, Mignardi M, Beausang J, Bottino R, Kim SK, Quake SR. 2017. Single-Cell Analysis of Human Pancreas Reveals Transcriptional Signatures of Aging and Somatic Mutation Patterns. Cell.*

*Martinez-Jimenez CP, Eling N, Chen HC, Vallejos CA, Kolodziejczyk AA, Connor F, Stojic L, Rayner TF, Stubbington MJT, Teichmann SA, et al. 2017. Aging increases cell-to-cell transcriptional variability upon immune stimulation. Science (80- ).*
