# Supplementary material for: Predicting lung aging using scRNA-Seq data
Source: PLoS Comput Biol. 2024 Dec 19;20(12):e1012632. doi: 10.1371/journal.pcbi.1012632 (PMC11741621; doi:10.1371/journal.pcbi.1012632)
Supplement: Attachment — Submitted filename: polyEN_response_letter_rev2_10-06-2024.docx [file pcbi.1012632.s008.docx]

*We wish to thank the reviewers for their constructive feedback to our revised manuscript. We provide our point-by-pint response below.*

**Reviewer #1:**

Authors addressed all my comments and I recommend to accept this manuscript.

*Thank you. We appreciate your effort for reviewing the manuscript.*

**Reviewer #2:**

To follow up on reviewer 1’s question: The methylation clocks work with bulk methylation data. Given, that the polyEN method’s performance gives no improvement when cell type information is combined, it would suggest that methylation clocks (bulk data) and polyEN (single-cell data) may not be directly comparable. A more direct comparison would be if all the single-cell gene expression was averaged across all cell types to create bulk information and see if transcriptome is still a better signal than methylation. Even if the donors were the same, the bulk and single-cell data would have very different distributions.

*The reviewer is correct that single cell methylation data may indeed lead to better results. However, unlike scRNA-Seq scMethyltion data is less common and so the fact that scRNA-Seq is useful for age predictions (and improves upon bulk methylation) is already an interesting finding in our view. So the comparison is still valid and informative. Still, following the reviewer suggestion we performed age prediction at pseudo-bulk level by averaging the expression across all cells in Nuclear-Seq data. We performed training and testing following the same steps as used in Fig 3B. Results, presented in Fig. 9S show that when using the best senescence marker lists (“Union” and “CellAge”), expression predicted chronological ages still slightly improves on methylation-based predictions. The improvement is indeed less significant than the single cell based predictions indicating that single cell data is more informative which is the overall theme of the paper. We added the relevant discussions in the main text, under the section titled “****Correlation between age prediction and methylation evidence****”.*

I had a really hard time understanding the response for comparing PolyEN with the elastic net model. It is unclear why the decision to present the ranking in Figure S7 was made instead of just presenting the R2 and RMSE value comparisons between the models. Could something like Figure 2(c) be plotted instead for this comparison?

*We appreciate the constructive feedback from the reviewer. As suggested we added R2 and RMSE values for the baseline elastic net model in Fig. 2C and Fig. S8. Results show that polyEN still significantly outperforms this baseline for nonsmokers (the larger group) but is less predictive than baseline elastic net model in smokers. We hypothesize that this may be due to the fact that smoking may accelerate the aging rate of airways as shown in Fig. S8 and in (1). Thus, younger smokers may have increased cell-cell variations similar to older non-smoker donors and given the limited number of individuals polynomial smoothing may mask these variations. We have updated the section titled “****PolyEN outperformed prior methods for predicting age from single cell RNA-seq data****” and ‘****Discussion****’ to mention this.*

The additional analysis of the SHAP values with coefficients is interesting. However, the observation that the top-ranked genes are different does not address the question completely. Was there a GO term analysis done for these genes like the one done for SHAP genes? Do these genes report similar GO terms or processes as SHAP genes? If they are different, what would that suggest?

*We thank the reviewer for clarifying the question raised in the first revision. We have added further analyses to address the follow-up questions. We performed GSEA analysis for coefficient-ranked genes, following the same steps that generate Fig. 5B. The results (Fig. S13) from the basal cells showed that the enriched terms are less relevant and interpretable, compared to enriched terms from genes identified by the SHAP-based method shown in Fig. 5. As for interpretation of the difference, we are not sure. It may be that the coefficient based approach can better sample from several different relevant processes making each less significant whereas the SHAP can identify several gene from the same process but miss others. But this is just a guess so we prefer not to mention it in the text. We added the results of this analysis to the section titled “****Apoptotic genes revealed by importance scores****”*

Also, is it possible that SHAP analysis is picking on highly expressed genes (looking at Figure 5 and 6)? If one were to rank the genes based on mean expression levels (or variance) and compare them to those ranked based on SHAP values, would one find significant overlap?

*We thank the reviewer for the suggestion. We performed the analysis accordingly and found there is a low overlap between gene ranking by highly expressed genes and gene ranking by SHAP-based method.* *we ranked genes by mean expressions in the three basal cell types shown in Fig 5. We observed very low overlap between the top-ranked gene sets from both methods. This shows that SHAP-based method has no strong bias towards highly expressed genes (Fig. S12). We have added the explanation into main text, under the section titled “****Apoptotic genes revealed by importance scores****”*

Finally, despite my recommendation to do a proofreading pass and authors’ response that this was done, I found several issues with the writing. See examples below:

“led to best results for smokers” in the last line reads abrupt and vague in the abstract.

*Thank you. We have rephrased the last sentence as “Our results identify lung epithelial cells as the most significant predictors for non-smokers while lung endothelial cells led to the best chronological age prediction results for smokers.”*

The author summary” has grammatical errors - “data profiling the gene expression”, “levels of genes in a individual”, “individual lung …. their ages”, “and that … as the best…”

*Thank you for pointing out the errors. We have corrected these in the revised manuscript.*

Line 52: “accumulated errors at various leves” reads a bit vague (what type of errors?).

*We meant different levels of the regulatory process. We now added the missing work. This is actually explicitly explained in the Introduction. “At the gene level, transcription error rate increases during cell senescence, leading to disease phenotypes. Additionally, telomere shortening and epigenetic regulation are also well-known molecular mechanisms modulating the cellular senescence processes. Particularly, epigenetic regulation is evidenced by strong correlation between DNA methylation changes and longevity of a wide range of organisms. At the cellular level, cell senescence is usually coupled with cell cycle arrest, characterized by stagnation in the G1 phase of the cell cycle . Cell communication is also known to be involved in this process. For example, the senescence-associated secretory phenotype (SASP) proteins secreted by cells can bind to surface receptors of surrounding cells and promote senescence in those cells. Various cytokines have also been suggested for playing a key role in promoting senescence through cell-cell interactions ”*

“P” capitalized in line 88 for polyEN but small in abstract.

*Thank you. We have corrected these in the revised manuscript.*

Line 157 “utilizes the following.” ends abruptly.

*Thank you. We have changed this to “utilizes the following steps to combine information from all cells” in the revised manuscript.*

Line 223 “Fleischer et al” needs a citation?

*Thank you. We have added the citation in the revised manuscript.*

“Overview of the analysis and datasets” and “Cell type mapping and dataset integration” should probably be in the Methods section and not Results.

*Adding a brief Methods overview at the beginning of Results is fairly common for biological journals since some readers skip Methods and go directly to Results. Since it’s a very brief repeat of the detailed information in Methods we kept it. As for the “Cell Type mapping and dataset integration” section, this section actually discusses results since it talks about successful and unsuccessful data integration for some of the datasets. We do have a more detailed sections on the methods used for integration (“Cell type transfer and data integration”) in Mehtods.*

Line 300: “P” caplitalized for p-value but small everywhere else.

*Thank you. We have corrected this in the revised manuscript.*

Line 297: “three previously published methods”.. is followed by listing only 2 - Fleischer et al and RAPTOR

*Thank you. We have changed it to “two previously published methods”*

*References*

1. *Walters,M.S., De,B.P., Salit,J., Buro-Auriemma,L.J., Wilson,T., Rogalski,A.M., Lief,L., Hackett,N.R., Staudt,M.R., Tilley,A.E., et al. (2014) Smoking accelerates aging of the small airway epithelium. Respir. Res., 10.1186/s12931-014-0094-1.*
